# Supplementary material for: Association of Glutathione Peroxidase 3 (GPx3) and miR-196a with Carbohydrate Metabolism Disorders in the Elderly
Source: Int J Mol Sci. 2024 May 15;25(10):5409. doi: 10.3390/ijms25105409 (PMC11121935; doi:10.3390/ijms25105409)
Supplement: Supplementary file 1 [file ijms-25-05409-s001.zip › ijms-2983183-supplementary.pdf]

Table S1. (**a**) Correlations between the level of GPx3 and miR-196a, anthropometric parameters, SBP and DBP in patients aged 65 and older from the control group (n=38), CMD (n=88), prediabetes (n=37) and T2DM (n= 51). (**b**) Correlations between the level of GPx3 and metabolic and renal parameters in patients aged 65 and older from the control group (n=38), with the CMD (n=88), with the prediabetes (n=37) and with T2DM (n=51).

(**a**)

	**Control (n=38)**		**CMD (n=88)**		**Prediabetes (n=37)**		**T2DM (n=51)**	
**Parameters**	**Rho**	**P**	**Rho**	**P**	**Rho**	**P**	**Rho**	**P**
miR-196a	0.212	0.202	0.174	0.110	0.163	0.349	0.113	0.430
Age [years]	-0.249	0.132	-0.009	0.937	0.011	0.947	-0.043	0.765
SBP (mmHg)	-0.274	0.096	-0.147	0.175	0.207	0.225	**-0.350**	**0.012**
DBP (mmHg)	**-0.321**	**0.049**	-0.046	0.673	0.201	0.240	-0.241	0.088
Body mass [kg]	0.051	0.762	-0.208	0.053	-0.263	0.121	-0.191	0.179
Height [m]	0.240	0.146	-0.191	0.076	-0.212	0.215	-0.191	0.181
BMI [kg/m2]	-0.187	0.261	-0.122	0.265	-0.123	0.475	-0.102	0.479
WC [cm]	0.026	0.879	-0.133	0.220	-0.216	0.205	-0.083	0.559
HC [cm]	-0.122	0.466	-0.020	0.854	-0.071	0.681	0.017	0.908
WHR	0.316	0.053	-0.151	0.164	0.015	0.929	-0.235	0.097
ST triceps (mm)	**-0.400**	**0.013**	-0.008	0.943	-0.029	0.866	0.047	0.744
ST abdominal (mm)	-0.092	0.584	-0.078	0.471	-0.107	0.535	-0.019	0.897
ST thigh (mm)	-0.163	0.329	-0.062	0.568	-0.144	0.402	0.066	0.644
Visceral Fat Rating	-0.149	0.371	-0.121	0.275	-0.182	0.295	-0.072	0.620
BIA – BF [%]	-0.213	0.199	0.086	0.439	-0.011	0.950	0.226	0.115
BIA - BF [kg]	-0.094	0.573	**-0.255**	**0.019**	-0.099	0.572	0.040	0.784
BIA - FFM [%]	0.218	0.188	-0.005	0.966	0.007	0.969	-0.266	0.062
BIA - FFM [kg]	0.184	0.268	**-0.234**	**0.031**	-0.213	0.220	-0.264	0.064
BIA-TBW [%]	0.304	0.063	-0.149	0.172	-0.089	0.612	**-0.283**	**0.046**
BIA-TBW [kg]	0.217	0.191	**-0.302**	**0.006**	-0.273	0.113	-0.228	0.111

(**b**)

	**Control (n=38)**		**CMD (n=88)**		**Prediabetes (n=37)**		**T2DM (n=51)**	
**Parameters**	**Rho**	**P**	**Rho**	**P**	**Rho**	**P**	**Rho**	**P**
HbA1c [%]	0.090	0.593	-0.119	0.273	0.074	0.668	-0.161	0.259
FPG [mmol/l]	0.095	0.572	**-0.291**	**0.006**	**-0.427**	**0.009**	-0.177	0.215
HOMA-IR	-0.011	0.947	0.110	0.309	-0.129	0.452	0.157	0.271
TG/ HDL ratio	-0.020	0.907	0.154	0.158	**0.337**	**0.044**	0.138	0.344
Creatinine [µmol/L]	**-0.470**	**0.003**	-0.095	0.383	0.079	0.646	-0.228	0.112
Urea [mmol/L]	-0.229	0.166	-0.211	0.051	-0.219	0.199	-0.174	0.227
eGFR [ml/min/1.73 m2]	**0.380**	**0.019**	0.007	0.950	-0.079	0.646	0.047	0.747
LDL [mmol/L]	0.230	0.164	0.023	0.834	-0.032	0.855	0.091	0.536
HDL [mmol/L]	-0.073	0.661	-0.117	0.283	**-0.377**	**0.023**	-0.043	0.768
TG [mmol/L]	-0.026	0.875	0.123	0.258	0.228	0.181	0.155	0.282
T-CH [mmol/L]	0.208	0.210	0.013	0.903	-0.147	0.391	0.099	0.496

List of abbreviations: T2DM—type 2 diabetic, BMI—body mass index, WHR—waist to hip ratio, WC—waist circumference, HC – hip circumference, ST -skinfold thickness , BIA- bioelectrical impedance analysis, BF-body fat, FFM- free fat mass- muscle mass,

TBW – total body water. Rho - Spearman's rank correlation coefficient. Statistically significant results are shown in bold. HbA1c—glycated hemoglobin FPG—fasting plasma glucose, HOMA-IR—homeostasis model assessment for insulin resistance, eGFR—estimated glomerular filtration rate, T-CH—total cholesterol, LDL-C—low-density lipoprotein cholesterol, HDL-C—high-density lipoprotein cholesterol, TG—triglycerides. Rho - Spearman's rank correlation coefficient. Statistically significant results are shown in bold.

Table S2. (**a**). Correlations between the level of miR-196a expression and anthropometric parameters, SBP and DBP in patients from the control group (n=38), CMD(n=88), prediabetes (n=37) and T2DM (n =51). (**b**) Correlations between the level of miR-196a expression and metabolic parameters in patients from the control group (n=38), CMD(n=88), prediabetes (n=37) and T2DM (n =51).

(**a**)

	**Control (n=38)**		**CMD (n=88)**		**Prediabetes (n=37)**		**T2DM (n=51)**	
**Parameters**	**Rho**	**P**	**Rho**	**P**	**Rho**	**P**	**Rho**	**P**
Age [years]	0.294	0.073	-0.141	0.191	-0.054	0.751	-0.164	0.249
SBP (mmHg)	-0.001	0.98	-0.049	0.649	-0.108	0.528	-0.017	0.904
DBP (mmHg)	-0.033	0.843	-0.041	0.702	-0.08	0.642	0.005	0.973
Body mass [kg]	-0.056	0.736	0.021	0.848	-0.036	0.832	-0.006	0.967
Height [m]	-0.092	0.579	-0.036	0.737	-0.051	0.769	-0.055	0.7
BMI [kg/m2]	0.104	0.532	0.078	0.474	-0.013	0.935	0.065	0.654
WC [cm]	-0.024	0.884	-0.008	0.938	-0.131	0.443	-0.018	0.896
HC [cm]	0.111	0.506	-0.052	0.627	-0.239	0.16	0.046	0.745
WHR	-0.046	0.782	0.007	0.946	0.027	0.875	-0.085	0.55
ST triceps (mm)	0.293	0.074	-0.016	0.877	-0.186	0.275	0.071	0.621
ST abdominal (mm)	-0.037	0.822	0.076	0.485	-0.043	0.803	0.043	0.765
ST thigh (mm)	0.137	0.412	0.119	0.271	0.053	0.757	0.099	0.486
Visceral Fat Rating	0.113	0.498	-0.0162	0.884	-0.004	0.984	-0.124	0.389
BIA – BF [%]	0.141	0.398	0.071	0.521	0.189	0.277	-0.120	0.408
BIA - BF [kg]	0.087	0.603	**0.251**	**0.021**	0.099	0.571	-0.065	0.649
BIA - FFM [%]	-0.178	0.284	-0.155	0.157	-0.181	0.297	0.14	0.331
BIA - FFM [kg]	-0.11	0.511	-0.02	0.855	-0.164	0.344	0.058	0.688
BIA-TBW [%]	-0.212	0.202	-0.043	0.692	-0.112	0.520	0.121	0.402
BIA-TBW [kg]	-0.172	0.3	0.191	0.084	-0.118	0.498	0.071	0.625

(**b**)

	**Control (n=38)**		**CMD (n=88)**		**Prediabetes (n=37)**		**T2DM (n=51)**	
**Parameters**	**Rho**	**P**	**Rho**	**P**	**Rho**	**P**	**Rho**	**P**
HbA1c [%]	-0.164	0.324	0.057	0.598	-0.157	0.361	0.016	0.910
FPG [mmol/l]	-0.008	0.959	-0.054	0.613	**-0.336**	**0.045**	-0.091	0.527
HOMA-IR	0.120	0.472	-0.045	0.677	-0.203	0.236	0.098	0.493
TG/ HDL ratio	-0.243	0.140	-0.083	0.449	0.009	0.956	-0.201	0.166
Creatinine [µmol/L]	0.239	0.148	0.026	0.809	-0.051	0.764	0.083	0.562
Urea [mmol/L]	0.165	0.321	-0.081	0.457	-0.003	0.985	-0.127	0.377
eGFR [ml/min/1.73 m2]	-0.139	0.404	-0.079	0.467	-0.061	0.722	-0.118	0.413
LDL [mmol/L]	**-0.499**	**0.001**	0.075	0.494	0.079	0.644	0.093	0.523

HDL [mmol/L]	0.109	0.515	0.084	0.438	0.133	0.440	0.121	0.400
TG [mmol/L]	-0.273	0.096	-0.024	0.823	0.180	0.291	-0.174	0.225
T-CH [mmol/L]	**-0.406**	**0.011**	0.039	0.715	0.137	0.424	0.001	0.992

List of abbreviations: T2DM—type 2 diabetic, BMI—body mass index, WHR—waist to hip ratio, WC—waist circumference, HC – hip circumference, ST -skinfold thickness , BIA- bioelectrical impedance analysis, BF-body fat, FFM- free fat mass- muscle mass, TBW – total body water. Rho - Spearman's rank correlation coefficient. Statistically significant results are shown in bold. HbA1c—glycated hemoglobin FPG—fasting plasma glucose, HOMA-IR—homeostasis model assessment for insulin resistance, eGFR—estimated glomerular filtration rate, T-CH—total cholesterol, LDL-C—low-density lipoprotein cholesterol, HDL-C—high-density lipoprotein cholesterol, TG—triglycerides. Rho - Spearman's rank correlation coefficient. Statistically significant results are shown in bold.

Table S3. Spearman correlations and linear regression analysis of Gpx3 concentration and anthropometric and biochemical parameters in the entire study population (n = 126). Only the best Gpx3-related variables are shown, as assessed by the stepwise forward multiple regression method.

	Whole study population (n=126)			
Parameters	Rho	P	β ± SE	P
MiR196a	0.165	0.067		
Age [years]	-0.128	0.155		
SBP (mmHg)	**-0.199**	**0.026**	**-0.190 ± 0.089**	**0.037**
DBP (mmHg)	-0.130	0.147		
Body mass [kg]	-0.112	0.214		
Height [m]	-0.012	0.898		
BMI [kg/m2]	-0.143	0.114		
HbA1c [%]	**-0.187**	**0.037**	-0.011 ± 0.167	0.948
FPG[mmol/l]	**-0.249**	**0.005**	-0.188 ± 0.166	0.262
HOMA-IR	0.115	0.200		
TG/ HDL ratio	0.080	0.381		
Creatinine [μmol/L]	**-0.200**	**0.026**	-0.133 ± 0.118	0.261
Urea [mmol/L]	**-0.242**	**0.007**	-0.024 ± 0.118	0.842
eGFR [ml.min.1,73m^2]	0.156	0.083		
LDL [mmol/L]	0.123	0.174		
HDL [mmol/L]	-0.074	0.412		
TG [mmol/L]	0.070	0.468		
T-CH [mmol/L]	0.092	0.309		
WC [cm]	-0.095	0.292		
HC [cm]	-0.044	0.630		
WHR	-0.030	0.744		
ST triceps (mm)	-0.140	0.119		
ST abdominal (mm)	-0.125	0.166		
ST thigh (mm)	-0.125	0.164		
Visceral Fat Rating	-0.159	0.081		
BIA – BF [%]	–0.041	0.654		
BIA - BF [kg]	**-0.268**	**0.003**	0.054 ± 0.091	0.558
BIA - FFM [%]	0.135	0.137		
BIA - FFM [kg]	-0.036	0.696		
BIA-TBW [%]	0.019	0.831		
BIA-TBW [kg]	-0.250	0.006		

List of abbreviations :BMI—body mass index, DBP—diastolic blood pressure, eGFR—estimated glomerular filtration rate, HDL-CH—HDL cholesterol, FPG—fasting plasma glucose, HbA1c—glycated hemoglobin, HC—hips circumference, LDL-CH—LDL

cholesterol, FPG—fasting plasma glucose, SBP—systolic blood pressure, T-CH—total cholesterol, TG—triglycerides, WC- waist circumference, WHR—waist-hip ratio. ST -skinfold thickness , BIA- bioelectrical impedance analysis, BF-body fat, FFM- free fat mass- muscle mass, TBW – total body water. ** Rho—Spearman's rank correlation coefficient. *** p-value. β ± SE ****—regression coefficient ± standard error. # variables were log-transformed prior to linear regression analysis The bolded results indicate statistically significant associations.
